# Adding free to total prostate-specific antigen levels in trials of prostate cancer screening

**DOI:** 10.1054/bjoc.1999.0988

**Published:** 2000-01-18

**Authors:** N J Wald, H C Watt, L George, P Knekt, K J Helzlsouer, J Tuomilehto

**Affiliations:** CRC Cancer Screening Group, Department of Environmental and Preventive Medicine, Wolfson Institute of Preventive Medicine, St Bartholomew's and the Royal London School of Medicine and Dentistry, Charterhouse Square, London, EC1M 6BQ, UK; National Public Health Institute, Mannerheimintie 166, Helsinki, 00300, Finland; Johns Hopkins University School of Hygiene and Public Health, Training Center for Public Health Research, Washington County Hospital, PO Box 2067, Hagerstown, 21742–2067, USA

**Keywords:** prostate-specific antigen, free prostate-specific antigen, clinical prostate cancer, population screening

## Abstract

We used a nested case–control design on data from men in four prospective studies (from the UK, Maryland in the USA, and two from Finland) with available stored serum samples to determine whether there was an advantage in measuring both free prostate-specific antigen (PSA) and total PSA as a potential screening test for prostate cancer. Of these men, 247 were verified through national vital statistics offices as having died of prostate cancer, or having developed the disease, and 953 men who did not develop prostate cancer (controls) were selected, matched to cases for age, study centre and sample storage duration. Fixing the false-positive rate at 1%, the prostate cancer detection rate (sensitivity) over the 3 years following serum collection (based on 14 cancers) increased from an estimated 95% using total PSA to 97% using free and bound PSA (that is, bound to α-antichymotrypsin which together with the free form is total PSA). Over a 6-year period (based on 41 cancers) a similar difference occurred (52% and 56% detection rates respectively). We conclude that there is no material advantage in adding free to total PSA in prostate cancer screening trials. © 2000 Cancer Research Campaign


British Journal of Cancer (2000) **82**(3), 731–736
© 2000 Cancer Research Campaign
DOI: 10.1054/ bjoc.1999.0988, available online at http://www.idealibrary.com on IDEAL®

### Adding free to total prostate-specific antigen levels in trials of prostate cancer screening

**NJ Wald[1], HC Watt[1], L George[1], P Knekt[2], KJ Helzlsouer[3] and J Tuomilehto[2]**

[1]CRC Cancer Screening Group, Department of Environmental and Preventive Medicine, Wolfson Institute of Preventive Medicine, St Bartholomew's and the Royal London School of Medicine and Dentistry, Charterhouse Square, London EC1M 6BQ, UK; [2]National Public Health Institute, Mannerheimintie 166, 00300 Helsinki, Finland; [3]Johns Hopkins University School of Hygiene and Public Health, Training Center for Public Health Research, Washington County Hospital, PO Box 2067, Hagerstown, MD 21742–2067, USA

**Summary** We used a nested case–control design on data from men in four prospective studies (from the UK, Maryland in the USA, and two from Finland) with available stored serum samples to determine whether there was an advantage in measuring both free prostate-specific antigen (PSA) and total PSA as a potential screening test for prostate cancer. Of these men, 247 were verified through national vital statistics offices as having died of prostate cancer, or having developed the disease, and 953 men who did not develop prostate cancer (controls) were selected, matched to cases for age, study centre and sample storage duration. Fixing the false-positive rate at 1%, the prostate cancer detection rate (sensitivity) over the 3 years following serum collection (based on 14 cancers) increased from an estimated 95% using total PSA to 97% using free and bound PSA (that is, bound to α-antichymotrypsin which together with the free form is total PSA). Over a 6-year period (based on 41 cancers) a similar difference occurred (52% and 56% detection rates respectively). We conclude that there is no material advantage in adding free to total PSA in prostate cancer screening trials. © 2000 Cancer Research Campaign

**Keywords:** prostate-specific antigen; free prostate-specific antigen; clinical prostate cancer; population screening

Prostate cancer is the second most common cause of death from cancer in men in the UK and the age-specific mortality from the disease is increasing. Prospective studies (using nested case–control design), including one previously carried out by us, have shown that the measurement of serum prostate-specific antigen (PSA) is an effective screening test for prostate cancer in healthy men (Ballentine Carter et al, 1992; Helzlsouer et al, 1992; Stenman et al, 1994; Gann et al, 1995; Whittemore et al, 1995; Parkes et al, 1995; Jacobsen et al, 1996).

PSA occurs in the blood mainly in a complex with $\alpha_1$-antichymotrypsin (PSA-ACT) and partly in its free form. Assays for (total) PSA detect both these forms, including the Hybritech assay used in our previous publication (Parkes et al, 1995) and the Wallac assay that we used in the study described in this paper which has been carried out on the same data set. The data of Stenman et al (1991) in symptomatic men suggested that measurement of PSA-ACT alone was more discriminatory than measurement of total PSA as a test for prostate cancer. Stenman et al (1994) also suggest that, in asymptomatic men with raised PSA concentrations, measurement of the ratio PSA-ACT to total PSA may improve discrimination between men who were subsequently diagnosed with prostate cancer and men who were not. Several subsequent studies suggested that use of the ratio will reduce the false-positive rate (that is, the proportion of men who do not develop prostate cancer with a positive test result, which is one minus specificity) in symptomatic men with raised PSA levels, at the expense of a small reduction in the detection rate (proportion of men developing prostate cancer with a positive test result, sensitivity), as summarized by Woodrum et al (1998). To clarify the issue in asymptomatic men using a large data set, we decided to measure free and total PSA in our stored serum samples in men who developed prostate cancer and in men who did not, and estimate the performance of screening using the different forms of PSA alone and in combination.

*Received 14 May 1999*
*Revised 4 August 1999*
*Accepted 10 August 1999*

*Correspondence to:* PNJ Wald

### METHODS

The study was based on the same four cohorts used in our previous publication (Parkes et al, 1995). In total there were 49 261 healthy men: the British United Provident Association (BUPA) study (London, UK) (Wald et al, 1987), the CLUE study in Washington County, Maryland (US) (Helzlsouer et al, 1992), the North Karelia project (Puska et al, 1979) and the Social Insurance Institution Mobile Clinic Health Survey (Knekt et al, 1988) (both in Finland), as previously described. Serum samples taken from healthy men on recruitment were frozen and stored, and 265 of these men (cases) with serum samples available subsequently developed clinical prostate cancer, or died of prostate cancer. A nested case–control study design was used. Controls were men from the same study who had not developed prostate cancer at the end of follow-up. Five controls were selected per case, except in the CLUE study (two controls selected per case), making a total of 1055. Controls were matched with each case for age (within 1 year), duration of storage of the sample (collected within the same calendar year) and the number of freeze–thaw cycles. Cases were followed up for a median of 17.5 years and controls for 17.4 years (range 10–20 years).

Total PSA was previously measured in the cases and controls using the Tandem-R PSA radioimmuno-assay kits (Hybritech) assay (Parkes et al, 1995). This study is based on 247 of the 265

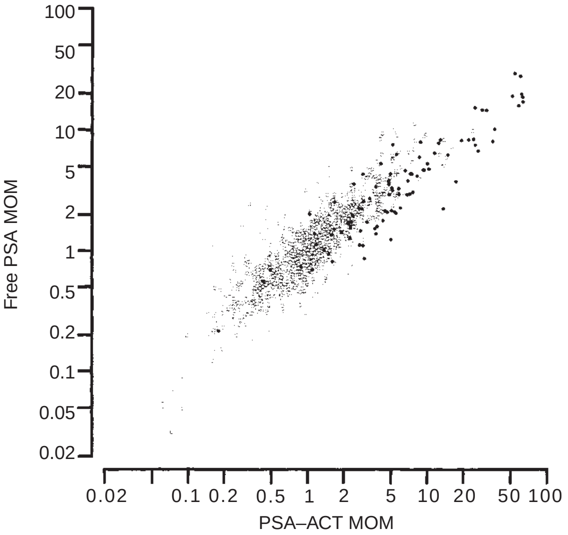

**Figure 1** Free PSA MOM values against PSA-ACT MOM values (adjusted for age and centre) in 953 men who did not develop prostate cancer (controls represented by open circles) and in 94 men who developed clinical prostate cancer within 10 years (solid circles)

cases and on 953 of the 1055 controls for whom a serum sample remained available for further assays. Samples from each case and control were retrieved from storage and assayed for total PSA and free PSA at the Wolfson Institute of Preventive Medicine, London, UK, without knowledge of which samples were from cases and which from controls. The Wallac PROSTATUS PSA free/total kit was used – an automatic immunoassay that uses the 1235 Auto DELFIA system for the simultaneous determination of total PSA and free PSA in serum. PSA-ACT was calculated by subtracting the free PSA concentration from the total PSA concentration.

Total PSA levels measured by the Wallac kit agreed very closely with measurements previously obtained using the Hybritech kit (correlation coefficient = 0.99); only the results using the Wallac assay are presented here.

The median concentrations of total PSA and free PSA in the controls were not significantly associated with duration of storage or number of freeze–thaw ‘cycles’. Total PSA, free PSA and PSA-ACT all increased with age (the increases per 10 years of age were 42% (95% confidence interval (CI) 28–57%), 51% (95% CI 37–67%) and 42% (95% CI 29–56%) respectively). To allow for this increase with age and for variations between centres, each concentration was expressed as a multiple of the median (MOM) for a given centre and age. The medians were derived from the controls using a weighted log-linear regression of median concentrations on age and centre. The median concentrations and median ages, in 5-year age groups, were taken for each centre, and weighted by the number of men in each group. A positive screening result was defined as a concentration of total PSA, PSA-ACT or free PSA that exceeded a specified MOM cut-off level. The performance of screening was assessed by fitting Gaussian distributions to the distributions of $\log_{10}$ MOM total PSA, free PSA, and PSA-ACT. A bivariate log Gaussian model was used to determine likelihood ratios for free PSA and PSA-ACT combined after confirming the range of values over which the data fitted log Gaussian distributions. This likelihood ratio was then applied to the overall risk of developing prostate cancer in the population, to give an estimated risk of developing prostate cancer for that individual man, which ignored the influence of age on his risk. Men with a risk estimate above a specified cut-off value were designated screen positive. The method has been published previously (Wald et al, 1988; Royston and Thomson, 1992).

### RESULTS

Figure 1 shows the MOM values of free PSA plotted against PSA-ACT MOM values in cases and controls. They were highly correlated (correlation coefficient 0.85 in controls, $P < 0.0001$). Because the correlation was high, one cannot have a substantially better screening performance than the other and both together can only make a modest improvement to the use of either alone.

Table 1 shows the median, 10th and 90th centiles of MOM total PSA, free PSA, and PSA-ACT in men who presented with clinical prostate cancer (cases) and in men who did not (controls). This shows that PSA-ACT, total PSA and free PSA were all high in cases (even in those who did not present until more than 10 years later).

Table 2 shows the detection and false-positive rates according to the different PSA measurements, different cut-off levels and different lengths of follow-up. The detection and false-positive rates were derived from fitting the Gaussian distribution to the distribution of MOM values. Probability plots of the three PSA measurements were reasonably linear (see Figure 2), showing that the three measurements were all approximately Gaussian. The plots also illustrate the extent of the separation in values between

**Table 1** Median (10th and 90th centiles) in multiples of the median (MOM) in controls of total PSA, free PSA and PSA-ACT in men who developed clinical prostate cancer (cases) according to time to diagnosis, and in men who did not (controls)

| Cases: time to presentation (years ) | No. of men | Total PSA | Free PSA | PSA-ACT[a] |
|---|---|---|---|---|
| <3 | 14 | 25 (8.3–53) | 9.1 (2.9–20) | 28 (8.8–61) |
| 3–5.9 | 27 | 4.7 (1.9–26) | 3.4 (1.4–14) | 4.8 (1.7–31) |
| 6–9.9 | 53 | 4.2 (1.1–9.4) | 2.1 (0.81–6.3) | 4.4 (1.1–10) |
| ≥10 | 153 | 1.9 (0.61–6.0) | 1.5 (0.52–4.6) | 2.0 (0.62–6.5) |
| All cases | 247 | 2.6 (0.75–11) | 1.8 (0.63–6.8) | 2.8 (0.73–12) |
| Controls | 953 | 0.98 (0.37–3.1) | 1.00 (0.39–2.8) | 0.98 (0.33–3.2) |

[a]Calculated by subtracting free PSA from total PSA and then converting into a MOM value.

© 2000 Cancer Research Campaign

**Table 2** Detection rates and false-positive rate of screening for clinical prostate cancer by measurement of total PSA, free PSA, and PSA-ACT standardized for age and centre, according to cut-off level and interval between blood sampling and diagnosis of prostate cancer, based on Gaussian distributions of risk factors

| | False-positive rate ($n$ = 953) | | | Detection rate | | | | | | | | |
|---|---|---|---|---|---|---|---|---|---|---|---|---|
| | | | | Within 3 years ($n$ = 14) | | | Within 6 years ($n$ = 41) | | | Within 10 years ($n$ = 94) | | |
| Cut-off level MOM[a] | Total PSA | Free PSA | PSA-ACT | Total PSA | Free PSA | PSA-ACT | Total PSA | Free PSA | PSA-ACT | Total PSA | Free PSA | PSA-ACT |
| ≥4 | 5.8 | 5.0 | 5.7 | 99.6 | 87 | 99.7 | 74 | 56 | 75 | 56 | 40 | 58 |
| ≥6 | 2.2 | 1.7 | 2.1 | 98 | 72 | 98 | 61 | 39 | 63 | 42 | 24 | 44 |
| ≥8 | 1.0 | 0.7 | 0.9 | 95 | 57 | 96 | 51 | 27 | 53 | 32 | 16 | 35 |
| ≥12 | 0.3 | 0.2 | 0.2 | 85 | 35 | 88 | 37 | 15 | 39 | 21 | 8 | 23 |

[a]MOM = multiples of the median value in men of the same age and at the same centre who do not develop clinical prostate cancer.

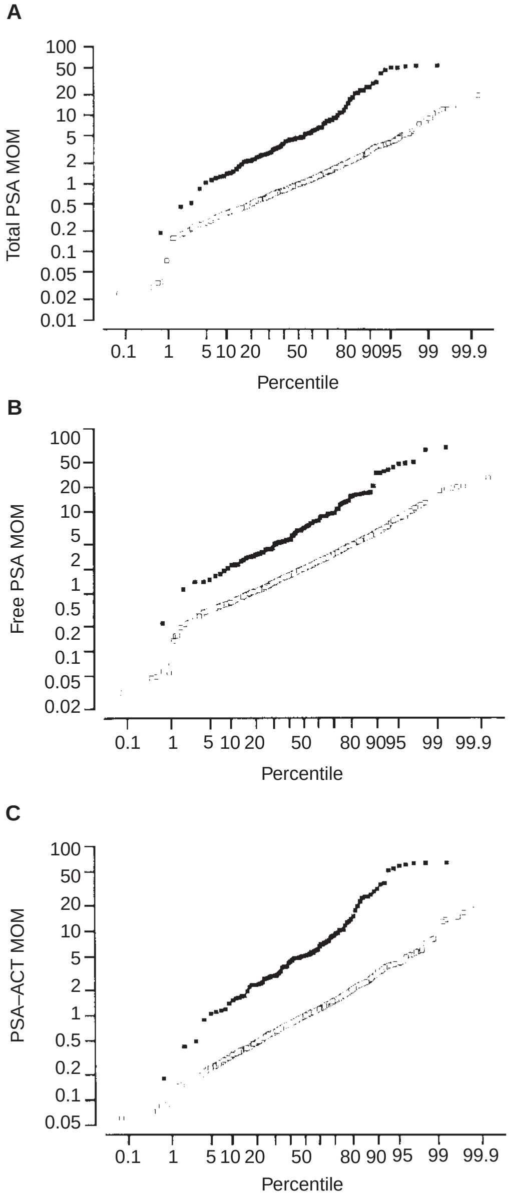

**Figure 2** Probability plot of total PSA (**A**), free PSA (**B**) and PSA-ACT (**C**) in multiples of the normal median (MOM) (log scale). Solid dots represent men who developed clinical prostate cancer within 10 years and open dots represent men who did not cases and controls. The false-positive rates at specified MOM cut-off levels were similar for the different tests. Total PSA and PSA-ACT were comparable in screening performance, and either was better than free PSA. Differences in the estimates in Table 2 compared with our earlier paper (Parkes et al, 1995) are small and due to the differences in the sample numbers and the fact that the present results were based on the model while the earlier ones were based on directly observed numbers.

Table 3 shows the detection rates according to specified false-positive rates, again based on the Gaussian distribution. This shows that free PSA and PSA-ACT combined give the highest detection rates, up to 2 percentage points higher for given false-positive rates than using PSA-ACT alone, and up to 4 percentage points higher than total PSA. Because PSA-ACT and free PSA are so highly correlated the two together are only modestly better in discriminating between affected and unaffected men than the better of the two (PSA-ACT) used alone.

Tables 5 and 6 give details of the cut-off levels used and the parameters of the distributions which will permit further statistical analysis of the results. Table 7 is a comparison of the detection rates based on the use of the Gaussian model with those directly observed by counting how many men had MOM values above specified levels. This shows good agreement between the two sets of estimates.

### DISCUSSION

Our results show that, were screening for prostate cancer shown to be worthwhile, there is only a small advantage in using free PSA and PSA-ACT combined appropriately rather than PSA-ACT alone or total PSA alone. For example, at a false-positive rate of 1%, the detection rate for cancers presenting within 3 years was 97% for free PSA and PSA-ACT combined, 96% for PSA-ACT and 95% for total PSA. The corresponding estimates for cancers presenting within 6 years were 56%, 54% and 52% respectively. If the cost of measuring PSA-ACT and free PSA is similar to measuring total PSA alone or PSA-ACT alone it may be worth taking advantage of the small increase in detection, but not otherwise.

We examined the value of measuring the ratio of PSA-ACT to total PSA in men with high serum total PSA levels (above 2 MOMs and above 4 MOMs), as previously recommended by Stenman et al (1991, 1994). Only men with both total PSA MOM values above the cut-off and a high ratio of PSA-ACT to total PSA were designated screen positive. This gave a comparable screening

*© 2000 Cancer Research Campaign* 

**Table 3** Detection rate of prostate cancer corresponding to specified false-positive rates for total PSA, free PSA, PSA-ACT, and for PSA-ACT and free PSA combined, standardized for age and centre, according to interval between blood sampling and diagnosis of prostate cancer. Performance determined by Gaussian modelling of the distributions

| | Detection rate | | | | | | | | | | | |
|---|---|---|---|---|---|---|---|---|---|---|---|---|
| | Within 3 years (*n* = 14) | | | | Within 6 years (*n* = 41) | | | | Within 10 years (*n* = 94) | | | |
| False-positive rate (%) | Total PSA | Free PSA | PSA-ACT | PSA-ACT and free PSA combined | Total PSA | Free PSA | PSA-ACT | PSA-ACT and free PSA combined | Total PSA | Free PSA | PSA-ACT | PSA-ACT and free PSA combined |
| 5.0 | 99.4 | 87 | 99.6 | 99.7 | 72 | 56 | 73 | 75 | 53 | 39 | 56 | 57 |
| 2.0 | 98 | 74 | 98 | 98.9 | 60 | 41 | 62 | 64 | 41 | 26 | 44 | 45 |
| 1.0 | 95 | 63 | 96 | 97 | 52 | 32 | 54 | 56 | 33 | 19 | 36 | 37 |
| 0.5 | 91 | 52 | 93 | 95 | 44 | 24 | 47 | 49 | 26 | 14 | 29 | 31 |

**Table 4** Median concentrations of total PSA, free PSA, and PSA-ACT in men who did not develop clinical prostate cancer (controls) according to age and centre

| | Men aged 50 Regressed median† | Men aged 45–54 Observed median | Men aged 60 Regressed median† | Men aged 55–64 Observed median |
|---|---|---|---|---|
| Total PSA | | | | |
| BUPA | 0.73 | 0.71 | 1.04 | 1.06 |
| CLUE | 0.61 | 0.70 | 0.87 | 0.85 |
| North Karelia | 0.60 | 0.59 | 0.86 | 0.78 |
| SIIMCHS | 0.62 | 0.64 | 0.88 | 0.82 |
| Free PSA | | | | |
| BUPA | 0.11 | 0.10 | 0.17 | 0.17 |
| CLUE | 0.15 | 0.17 | 0.22 | 0.20 |
| North Karelia | 0.13 | 0.14 | 0.19 | 0.17 |
| SIIMCHS | 0.14 | 0.15 | 0.22 | 0.20 |
| PSA-ACT | | | | |
| BUPA | 0.61 | 0.58 | 0.87 | 0.88 |
| CLUE | 0.48 | 0.49 | 0.68 | 0.66 |
| North Karelia | 0.47 | 0.48 | 0.67 | 0.62 |
| SIIMCHS | 0.45 | 0.46 | 0.64 | 0.62 |

[a]The regressed medians were constrained to have the same percentage increase in concentrations per 10 years of age for each centre. BUPA – British United Provident Association, London, UK. CLUE – Washington County, MD, USA. North Karelia – Finland. SIIMCHS – Social Insurance Institution Mobile Clinic Health Survey, Finland.

**Table 5** Cut-off levels in multiples of the normal median (MOM) concentrations in control men of the same age and at the same centre corresponding to specified false-positive rates for total PSA, free PSA and PSA-ACT measurement. Men with MOM values above the specified cut-off level are designated screen positive. Calculation based on Gaussian distributions of risk factors

| | Cut-off level | | |
|---|---|---|---|
| False-positive rate (%) | Total PSA | Free PSA | PSA-ACT |
| 5.0 | 4.3 | 4.2 | 4.0 |
| 2.0 | 6.2 | 6.1 | 5.7 |
| 1.0 | 7.9 | 7.8 | 7.1 |
| 0.5 | 9.8 | 9.7 | 8.8 |

performance for a given false-positive rate, compared with using total PSA alone.

Following previous practice, we expressed the concentration in three forms of PSA in MOMs for men who did not develop prostate cancer who were of the same age and from the same centre. This conveniently allows for the increase in concentrations with age, and differences that can arise from centre to centre. Table 4 shows the observed and regressed (or expected) medians according to age and centre, which illustrates the importance of correcting for these effects. The regressed median concentrations correspond to MOM values of one.

Analysis of our data, based on the variation in duration of storage of the samples (between 15 and 22 years), shows that total PSA is stable but free PSA declines at about 2% per year ($P$ = 0.06). Previous published studies also show a decline in free PSA during frozen storage (Woodrum et al, 1996, 1998; Wu and Stephenson, 1997). There is an additional small loss of PSA when the blood sample is stored at room temperature before centrifugation, of 0.5–1.0% per h (Piironen et al, 1996; Woodrum et al, 1996) and when serum is stored before freezing, of 3–4% per day (Woodrum et al, 1996). After freezing, freeze–thaw cycles do not cause detectable loss of free PSA concentration (Piironen et al, 1996; Woodrum et al, 1996). The average free PSA concentration in the control samples in our study (mean 0.27 $\mu g\ L^{-1}$, standard deviation 0.34 $\mu g\ L^{-1}$) was about 30% lower than values in men in the same age range based on measurements in fresh samples (Orstein et al, 1997; Lein et al, 1998) and this is consistent with the estimated 2% per year loss during frozen storage and the small loss before freezing.

This loss of free PSA will not affect our estimates of screening performance. There is no reason to expect that the proportionate

 *© 2000 Cancer Research Campaign*

**Table 6** Means, standard deviations and correlation coefficients of total PSA, free PSA and PSA-ACT in men who did and did not develop clinical prostate cancer

| | Men who did not develop clinical prostate cancer | Men who developed clinical prostate cancer | | |
|---|---|---|---|---|
| | | Within 3 years | Within 6 years | Within 10 years |
| Means ($log_{10}$ MOM) | | | | |
| Total PSA | –0.0081 | 1.3949 | 0.9180 | 0.6718 |
| Free PSA | 0.0010 | 0.9578 | 0.6638 | 0.4927 |
| PSA-ACT | –0.0074 | 1.4421 | 0.9429 | 0.7017 |
| Standard deviations ($log_{10}$ MOM) | | | | |
| Total PSA | 0.3884 | 0.3018 | 0.4870 | 0.4962 |
| Free PSA | 0.3663 | 0.3133 | 0.3978 | 0.4106 |
| PSA-ACT | 0.3862 | 0.3092 | 0.5083 | 0.5155 |
| Correlation coefficients ($log_{10}$ MOM) | | | | |
| PSA-ACT, free PSA | 0.8502 | 0.8069 | 0.9190 | 0.9180 |

**Table 7** Comparison of performance of screening for clinical prostate cancer as determined by Gaussian modelling of the distributions (expected) to that observed directly by counting the number of men with MOM values beyond the cut-off levels. Detection rates of clinical prostate cancer diagnosed within 10 years corresponding to specified false-positive rates for total PSA, free PSA and PSA-ACT, standardized for age and centre

| | Detection rate (%) | | | | | |
|---|---|---|---|---|---|---|
| | Total PSA | | Free PSA | | PSA-ACT | |
| False-positive rate (%) | Observed (95% CI) | Expected | Observed (95% CI) | Expected | Observed (95% CI) | Expected |
| 5.0 | 60 (49–70) | 53 | 39 (29–50) | 39 | 62 (51–72) | 56 |
| 2.0 | 39 (24–56) | 41 | 26 (17–36) | 26 | 41 (32–53) | 44 |
| 1.0 | 27 (18–37) | 33 | 13 (6–21) | 19 | 30 (21–40) | 36 |
| 0.5 | 21 (14–31) | 26 | 12 (6–20) | 14 | 20 (13–30) | 29 |

loss would be different, on average, between the men who developed prostate cancer and those who did not, or to expect any material variation in proportionate loss between individuals in view of the high correlation of free PSA with both total PSA and PSA-ACT ($r$ = 0.85) in our data (Figure 1). With a constant proportionate loss, the ranking of samples would be preserved and estimated detection rates for given false-positive rates would, therefore, be unchanged.

While the performance of either total PSA or free PSA and PSA-ACT together as a screening test for future clinical prostate cancer is good, the value of screening for prostate cancer is unknown because the efficacy and morbidity of treatment are unknown. Early treatment of asymptomatic men would need to be better than treating men once disease had been clinically diagnosed. Side-effects from early treatments and psychological effects of early diagnosis would need to be balanced against any improvements in life expectancy. Screening has already been implemented and partially assessed in some places in the USA (Arcangeli et al, 1997) in the absence of evidence of benefit. The results from randomized screening trials that are underway are needed to assess the presence of benefit (Auvinen et al, 1996).

### ACKNOWLEDGEMENTS

We are grateful to Dr Malcolm Law for his comments on this manuscript. We thank Wallac Oy for providing the reagents for the PSA measurements. We thank the BUPA Foundation and the Cancer Research Campaign for their financial support.

### REFERENCES

Arcangeli CG, Ornstein DK, Keetch DW and Andriole GL (1997) Prostate-specific antigen as a screening test for prostate cancer. *Urol Clin North Am* **24:** 299–306

Auvinen A, Rietbergen JBW, Denis LJ, Schröder FH, Prorok PC for the International Prostate Cancer Screening Trial Evaluation Group (1996) Prospective evaluation plan for randomised trials of prostate cancer screening. *J Med Screening* **3:** 97–104

Ballentine Carter H, Pearson JD, Metter J, Brant LJ, Chan DW, Andres R, Fozard JL and Walsh PC (1992) Longitudinal evaluation of prostate specific antigen levels in men with and without prostate disease. *JAMA* **267**: 2215–2220

Gann PH, Hennekens CH and Stampfer MJ (1995) A prospective evaluation of plasma prostate-specific antigen for detection of prostatic cancer. *JAMA* **273**: 289–294

Helzlsouer KJ, Newby J and Comstock GW (1992) Prostate-specific antigen levels and subsequent prostate cancer: potential for screening. *Cancer Epidemiol Biomarkers* **1**: 537–540

Jacobsen SJ, Bergstralh EJ, Guess HA, Katusic SK, Klee GG, Oesterling JE and Lieber MM (1996) Predictive properties of serum prostate specific antigen testing in a community-based setting. *Arch Int Med* **156**: 2462–2468

Knekt P, Aromaa A, Maatela J, Alfthan G, Aaran RK, Teppo L and Hakama M (1988) Serum vitamin E, serum selenium and the risk of gastrointestinal cancer. *Int J Cancer* **42**: 846–850

Lein M, Koenig F, Jung K, McGovern FJ, Skates SJ, Schnorr D and Loening SA (1998) The percentage of free prostate specific antigen is an age-independent tumour marker for prostate cancer: establishment of reference ranges in a large population of healthy men. *Br J Urol* **82**: 231–236

Orstein DK, Snith DS, Rao GS, Basler JW, Ratliff TL and Catalona WJ (1997) Biological variation of total, free and percent free serum prostate specific antigen levels in screening volunteers. *J Urol* **157**: 2179–2182

Parkes C, Wald NJ, Murphy P, George L, Watt HC, Kirby R, Knekt P, Helzlsouer KJ and Tuomilehto J (1995) Prospective observational study to assess value of prostate specific antigen as screening test for prostate cancer. *Br Med J* **311**: 1340–1343

© 2000 Cancer Research Campaign 

Piironen T, Pettersson K, Suonpaa M, Stenman UH, Oesterling JE, Lovgren T and Lilja H (1996) In vitro stability of free prostate specific antigen (PSA) and prostate specific antigen (PSA) complexed to $\alpha_1$-antichymotrypsin in blood samples. *Urology* **48**: 81–87

Puska P, Tuomilehto J, Salonen JT, Neittaanmaki L, Maki J, Virtamo J, Nissinen A, Koskela K, Takalo T (1979) Changes in coronary risk factors during comprehensive 5-year community programme to control cardiovascular diseases (the North Karelia Project). *Br Med J* **ii**: 1173–1178

Royston P and Thompson SG (1992). Model-based screening by risk with application to Down's syndrome. *Stat Med* **11**: 257–268

Stenman UH, Hakama M, Knekt P, Aromaa A, Teppo L and Leinonen J (1994) Serum concentrations of prostate specific antigen and its complex with $\alpha_1$-antichymotrypsin before diagnosis of prostate cancer. *Lancet* **344**: 1594–1598

Stenman UH, Leinonen J, Alfthan H, Ranniko S, Tuhkanen K and Alfthan O (1991) A complex between prostate specific antigen and $\alpha_1$-antichymotrypsin is the major form of prostate specific antigen in serum of patients with prostatic cancer: assay of the complex improves clinical sensitivity for cancer. *Cancer Res* **51**: 222–226

Wald NJ, Thompson SG, Densem JW, Boreham J and Bailey A (1987) Serum vitamin E and subsequent risk of cancer. *Br J Cancer* **56**: 69–72

Wald NJ, Cuckle HS, Densem JW, Nanchahal K, Royston P, Chard T, Haddow JE, Knight GJ, Palomaki GE and Canick JA (1998) Maternal serum screening for Down's syndrome in early pregnancy. *Br Med J* **297** 883–887

Whittemore AS, Lele C, Friedman GD, Stamey T, Vogelman JH and Orentreich N (1995) Prostate-specific antigen as predictor of prostate cancer in black men and white men. *J Natl Cancer Inst* **87** 354–360

Woodrum D, French C and Shamel B (1996) Stability of free prostate specific antigen in serum samples under a variety of sample collection and sample storage conditions. *Urology* **48** 33–39

Woodrum DL, Brawer MK, Partin AW, Catalona WJ and Southwick PC (1998) Interpretation of free prostate specific antigen clinical research studies for the detection of prostate cancer. *J Urol* **159** 5–12

Wu JT and Stephenson RA (1997) Microplate assay for free PSA, PSA ACT and total PSA: assay characteristics, selection of calibrators and in vitro stability studies. In: *First Consultation on Prostate Cancer*, Murphy G, Griffiths K, Denis L, Khoury S, Chatelain C and Crockett AT (eds). Scientific Communication International: London

 *© 2000 Cancer Research Campaign*
